# When distance matters: Mapping HIV health care underserved communities in sub-Saharan Africa

**DOI:** 10.1371/journal.pgph.0000013

**Published:** 2021-11-24

**Authors:** Hana Kim, Godfrey N. Musuka, Zindoga Mukandavire, Adam Branscum, Diego F. Cuadros

**Affiliations:** 1 Department of Geography and Geographic Information Science, University of Cincinnati, Cincinnati, Ohio, United States of America; 2 Health Geography and Disease Modeling Laboratory, University of Cincinnati, Cincinnati, Ohio, United States of America; 3 ICAP at Columbia University, Harare, Zimbabwe; 4 Centre for Data Science and Artificial Intelligence, Emirates Aviation University, Dubai, UAE; 5 Department of Biostatistics, College of Public Health and Human Sciences, Oregon State University, Corvallis, Oregon, United States of America; The University of Alabama, UNITED STATES

## Abstract

Despite efforts to increase the proportion of individuals diagnosed with HIV who receive anti-retroviral therapy, 28% of people living with HIV (PLHIV) aged 15 years and older in eastern and southern Africa and 42% in western and central Africa were not receiving anti-retroviral therapy in 2019. Therefore, improving access to health care services is key to reduce HIV incidence and prevalence. The main aim of this study was to generate high-resolution maps of underserved areas where people cannot access the closest health care facilities within appropriate travel time in sub-Saharan Africa (SSA). Main sources of data for this study were the estimated number of PLHIV for adults aged 15–49 years in 47 countries in SSA and the global map of travel time to the nearest health care facility by motorized and non-motorized transportation. These data were used to estimate and map the number of PLHIV in underserved areas at a travel distance of 10, 30, and 60 minutes from the nearest healthcare facility. We identified and mapped more than 7 million PLHIV in the areas with a lack of access to health care within 10-minute travel time and 1.5 million PLHIV in the areas with a lack of access to health care within 60-minute travel time. The identified locations of underserved areas are an indicator of the challenge faced by PLHIV in accessing health services in SSA, a situation that is likely worsened by the COVID-19 pandemic. These findings can contribute to developing cost-effective geospatial policies for interventions aimed at underserved areas at a finer resolution for communities that have usually been identified in aggregated spatial areas. Further development and implementation of tailored intervention and treatment programs, especially in areas identified as underserved for PLHIV, should be explored. Geospatial analyses could complement the decision-making process with stakeholders to enhance healthcare access for PLHIV in SSA.

## Introduction

HIV/AIDS has been a leading cause of morbidity and mortality for several decades in sub-Saharan Africa (SSA) [1–3]. In 2019, SSA had the highest number of new HIV infections, HIV prevalence, and people living with HIV (PLHIV) in the world [4]. Despite efforts to increase the proportion of individuals diagnosed with HIV who receive anti-retroviral therapy, 28% (95% confidence interval [CI]: 19–38%) of PLHIV aged 15 years and older in eastern and southern Africa and 42% (95% CI: 25–56%) of PLHIV aged 15 years and older in western and central Africa were not receiving anti-retroviral therapy in 2019 [5, 6]. In this context, improving access to health care services is key to reduce HIV incidence and prevalence [2, 3].

In 2015, the Joint United Nations Programme on HIV/AIDS (UNAIDS) established the 90-90-90 global targets (90% of all PLHIV know their HIV status, 90% of all people diagnosed with HIV receive sustained antiretroviral therapy, and 90% of all people receiving antiretroviral therapy have viral suppression) by 2020 for HIV treatment scale-up to end AIDS by 2030. This move highlighted that uninterrupted and expanded access to treatment via rapid scale-up is crucial for PLHIV, especially in resource-limited settings [3]. Some African countries, such as Rwanda, Uganda, Zimbabwe, and Zambia, have met the target toward elimination of AIDS by 2030 [2, 6]. However, the current global target, which expired at the end of 2020, was largely not achieved due in part to the resources needed to address emerging health challenges like the 2019 novel coronavirus (COVID-19) [2, 3]. As a result, UNAIDS proposed new targets for 2025 in the Sustainable Development Goal 3.3 aimed at ending the AIDS epidemic by 2030 [2]. The 2025 targets have three categories: comprehensive HIV services, people-centered, context-specific service integration, and removal of societal and legal impediments to create a conducive environment for HIV services [2]. Besides the original goals (95% of PLHIV know their HIV status, 95% of PLHIV who know their status on treatment, and 95% on treatment are virally suppressed) by 2030, UNAIDS set additional goals for 2025 focusing on increasing access to HIV services for PLHIV and other key population groups to eliminate AIDS by 2030. 90% of PLHIV and people at risk are linked to people-centered and context-specific integrated services; 95% use combination prevention, women access health services, and coverage of services for eliminating vertical transmission; less than 10% experience stigma and discrimination, and gender inequality and violence. Identifying financial, social, and geographic barriers that impede HIV service access is key to achieve the 2025 targets. Specifically, removal of geographic barriers (e.g., proximity to health care for populations at high risk) and improving access to health services are vital to maintain essential health services and mitigate the impact of COVID-19 for PLHIV [2].

HIV is a chronic disease, and thus continuous access to health services for PLHIV is extremely important. This consideration emerged as a result of improvements to advanced treatments and the nature of the current HIV epidemic, namely reduced incidence of HIV and a larger proportion of people on antiretroviral therapy [7]. Seeing a doctor regularly for monitoring and/or treatment of HIV has become a part of life for PLHIV, thus health care facilities within accessible travel time are important.

With an increased focus on access to health care services and to meet the global targets, studies for measuring access to health care in SSA have shown that spatial analysis is an effective methodology to characterize accessibility to health care facilities [8–15]. In SSA, access to health care is measured using travel time for the general population [8–12] as potential patients, maternal health services [13], and public facilities for fever treatment [14]. Identifying areas where health care services cannot be accessed within appropriate travel time and identifying vulnerable populations at high risk of infection need to be prioritized to meet global targets for 2025. However, most past measures of accessibility in SSA are frequently available only for large geographical administrative units (i.e., low spatial resolution), such as at the national or sub-national level. These large-scale measures simplify heterogeneous spatial patterns within each country and are therefore not suitable for designing spatially targeted interventions. In addition, previous studies on accessibility in SSA have not focused on PLHIV, and high-resolution analysis of access to health care for PLHIV has not been considered. Microlevel analyses on the underserved areas and the locations of PLHIV within these underserved areas could help prevent new HIV infections and increase treatment access by prioritizing certain areas within local communities.

Against this background, the main aim of this study is to generate high-resolution maps of underserved areas where people cannot access the closest health care facilities within appropriate travel time in SSA. In addition, spatial estimates are used to identify PLHIV residing in the areas with low access to interventions for HIV prevention and treatment. This information can become essential for informing national HIV strategies to meet UNAIDS targets in SSA.

## Methods

### Study area and data sources

The main sources of data for this study were twofold: the estimated number of PLHIV for adults aged 15–49 years in 47 countries in SSA and the global map of travel time to the nearest health care facility by motorized and non-motorized transportation [9, 16]. The estimated number of PLHIV for adults aged 15–49 years was generated by combining these two different sources of data, the estimated surface of HIV prevalence and the population density surface, both in raster form [1, 16, 17]. Briefly, the HIV prevalence surface was calculated using a Bayesian spatiotemporal generalized linear mixed effects model using 13 covariates by considering possible nonlinear effects and complex interactions. The estimated HIV prevalence raster was then multiplied by the corresponding population estimate raster from WorldPop to estimate number of PLHIV in each 5km x 5km pixel across 47 countries in SSA [16, 17] using the raster calculator in ArcGIS [18]. We obtained the final estimates of PLHIV in 2017 in 5km x 5km resolution from the Global Health Data Exchange [16, 19]. Further details related to this dataset can be found in S1 Text.

The global map of travel time to the nearest health care facility by motorized and non-motorized transportation was generated in [2] to represent the spatial heterogeneity of geographically constrained healthcare access experienced by individuals with access to motorized transportation [8]. A database was created in [9] based on health care facility locations and friction surfaces that quantified the time required to traverse each grid within a 1km x 1km pixel resolution of the Earth’s surface. Travel times to the nearest health care facility were calculated at each pixel by considering two types of human movement: optimal, mostly motorized transportation, and walking-only. For the travel time from optimal friction surface, the speed of human movement is assumed as the motorized movement along roadways, railways and on water. For the travel time from walking-only friction surface, the speed of human movement is assumed as reduced walking speed (5km per hour on all roads and 1km per hour on wetland and water). A least-cost-path algorithm was employed to calculate the travel time [20]. All possible routes from each pixel were calculated to every health care facility and the minimum travel time for each pixel is displayed. We obtained the final friction surfaces of travel time to hospitals and clinics at 1km x 1km resolution from the Malaria Atlas Project (MAP) [9, 20]. Further details of the dataset are presented in S1 Text.

We extracted the datasets for Africa corresponding to the areas of PLHIV for adults aged 15–49 years to generate the density of PLHIV in underserved areas in Africa. Administrative boundaries of all countries were retrieved from the Database of Global Administrative Areas (GADM) dataset as a shapefile implemented by the Center for Spatial Sciences at the University of California, Davis [21].

### Mapping PLHIV in underserved areas

To identify PLHIV in underserved health care areas, we reduced the resolution of the maps of travel time to health care facilities from 1km x 1km pixel to 5 km x 5 km pixel for consistency with the resolution of the density map of PLHIV in Africa. The travel time surface was reclassified into a Boolean surface based on whether the cell was greater than three thresholds, 10, 30, and 60 minutes, from a health care location. All cells within a threshold were classified as 1, and all others as 0. Then, we converted the underserved areas that are classified as 1 from a raster grid to a vector (polygons) to identify the density of PLHIV based on the vector boundary. Finally, we generated density maps of PLHIV in the identified underserved areas by extracting continuous surface maps of PLHIV within each threshold underserved area boundary. Detailed descriptions of threshold times can be found S1 Text.

In addition, we used zonal statistics, which calculates the values within the defined zone, to calculate the number of PLHIV within each underserved area for each administrative unit, such as country boundary. Briefly, the zonal statistics is a commonly used spatial analysis procedure for grid data that calculates statistics on values of each grid within the zone defined by another dataset (for this study, the specific travel time). We used zonal statistics to sum the number of PLHIV in each pixel within the underserved areas by country. For example, if we calculate the number of PLHIV within underserved areas with 30-minute threshold travel time in a given country, the number of PLHIV within each pixel within the zone were calculated. From the resulting counts, we calculated the proportion of PLHIV within underserved areas out of the total PLHIV in the country. For each country, we generated cumulative bar graphs of the proportion of PLHIV within each underserved area for each country, and the proportion changes at the threshold time level in each country. Lastly, we generated Lorenz curves of the cumulative numbers of PLHIV at the 5 km x 5 km pixel level. Briefly, Lorenz curves, which were derived from Economics to access wealth inequality, depict the degree of spatial distribution by showing the cumulative proportion of PLHIV (x-axis) against the cumulative proportion of 5km x 5km pixel in the study area (y-axis). Lorenz curve is generally convex to the y-axis, of which the upper limit is uniform distribution line when *y = x*. We applied the Lorenz curve to graphical and quantitative indicators of inequality to provide a qualitative representation of the degree of inequality by showing the cumulative proportion of PLHIV against the cumulative proportion of 5km x 5km pixel in the study area. We showed the relationship between number of PLHIV and their occupied areas in a curve with a concave slope, and the curve *y = x* is the perfect equality meaning PLHIV equally reside within the areas (pixel).

## Results

### PLHIV within underserved areas with motorized transportation

**Fig 1:**
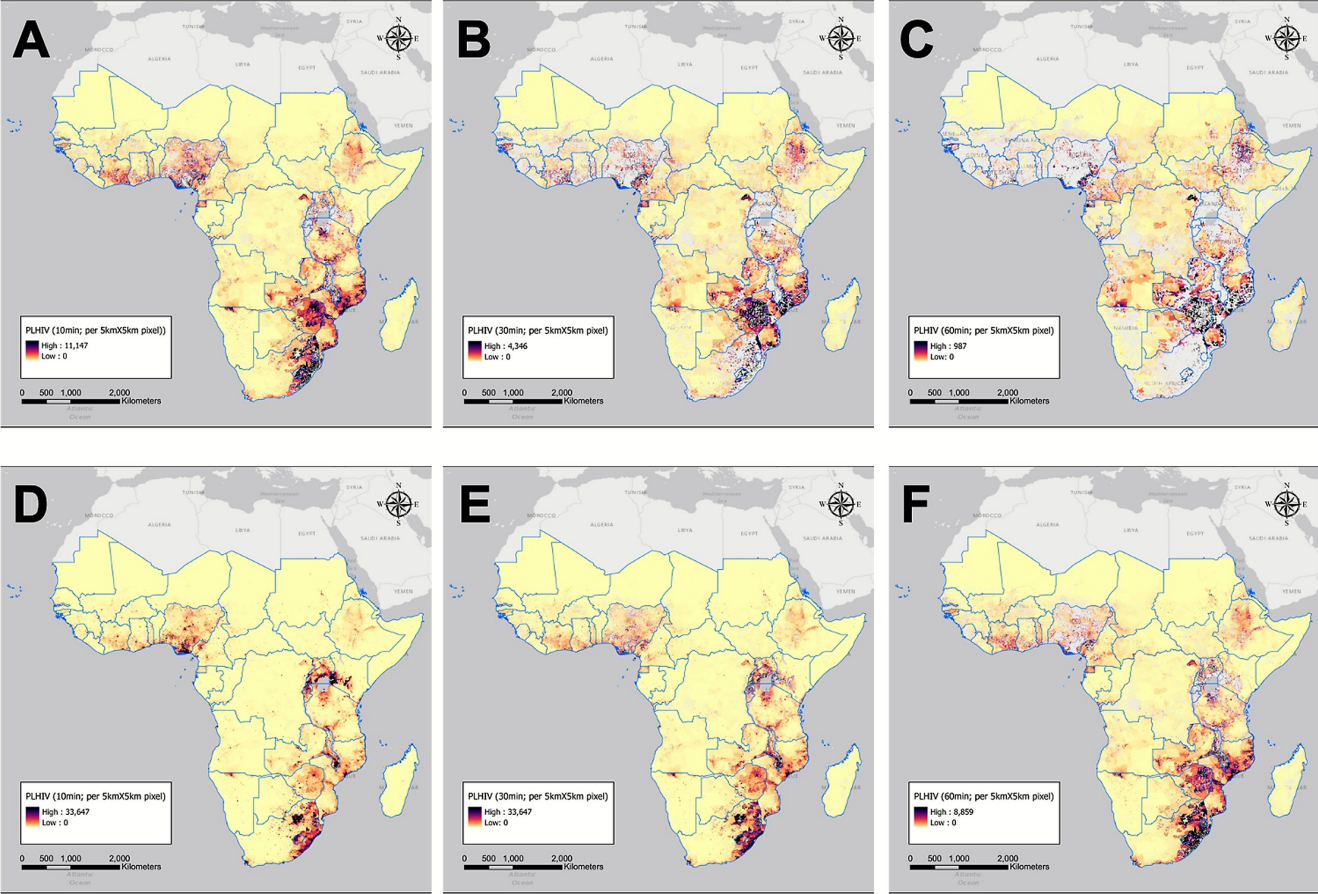
People Living with HIV (PLHIV) in areas with underserved access to health care in sub-Saharan Africa. PLHIV with access to the nearest health care facility using motorized or non-motorized transport at the 10-minute threshold (A. motorized; D. non-motorized), 30-minute threshold (B. motorized and E. non-motorized), and 60-minute threshold (C. motorized; F. non-motorized). Maps were created using ArcGIS by ESRI version 10.5 (http://www.esri.com) [18].

Fig 1A–1C illustrate the spatial distribution of the estimated number of PLHIV with access to motorized transportation in underserved areas with 10-, 30-, and 60-minute travel times as a threshold by 5km x 5km pixel. Similar spatial patterns of PLHIV in underserved areas were observed for all thresholds, showing high density of PLHIV in south-eastern areas. The underserved areas based on 10-minute travel time to the nearest health care facility across the region covered about 90.5% of the total territory (≈ 21.8M km^2^; Fig 1A), while 74.6% (Fig 1B) and 58.9% (Fig 1C) of SSA had underserved areas that were within 30- and 60-minute thresholds, respectively. These areas corresponded to 35% (≈ 7M), 15.6% (≈ 3M), and 7.6% (≈ 1.5M) PLHIV of the total PLHIV aged 15–49 in SSA, respectively. Descriptions of underserved areas at the country level can be found in S1 Text.

As the threshold time increased from 10 to 60 minutes, the average proportion of underserved areas decreased from 80.9% to 42.6% in SSA. Specifically, underserved areas in some countries such as Zimbabwe, Somalia, Equatorial Guinea and Mozambique, were significantly reduced from about 90% to less than 50% (95.4% to 43%; 95.1% to 48.7%; 95% to 43.9%; and 93.6% to 49%, respectively). On the other hand, underserved areas, which covered more than 50% of the territory with a 10-minute threshold, reduced to less than 10% with a 60-minute threshold in some countries (e.g., Sierra Leone, Gambia, and Swaziland; 56.9% to 6.5%, 55.8% to 2.8%, and 50.6% to 1.4%, respectively). In Sudan and Mauritania, 99.4% of areas were underserved at the 10-minute threshold, and more than 90% of areas were underserved at the 60-minute threshold (99.4% to 93.3%, and 99.4% to 91.2%, respectively).

**Fig 2:**
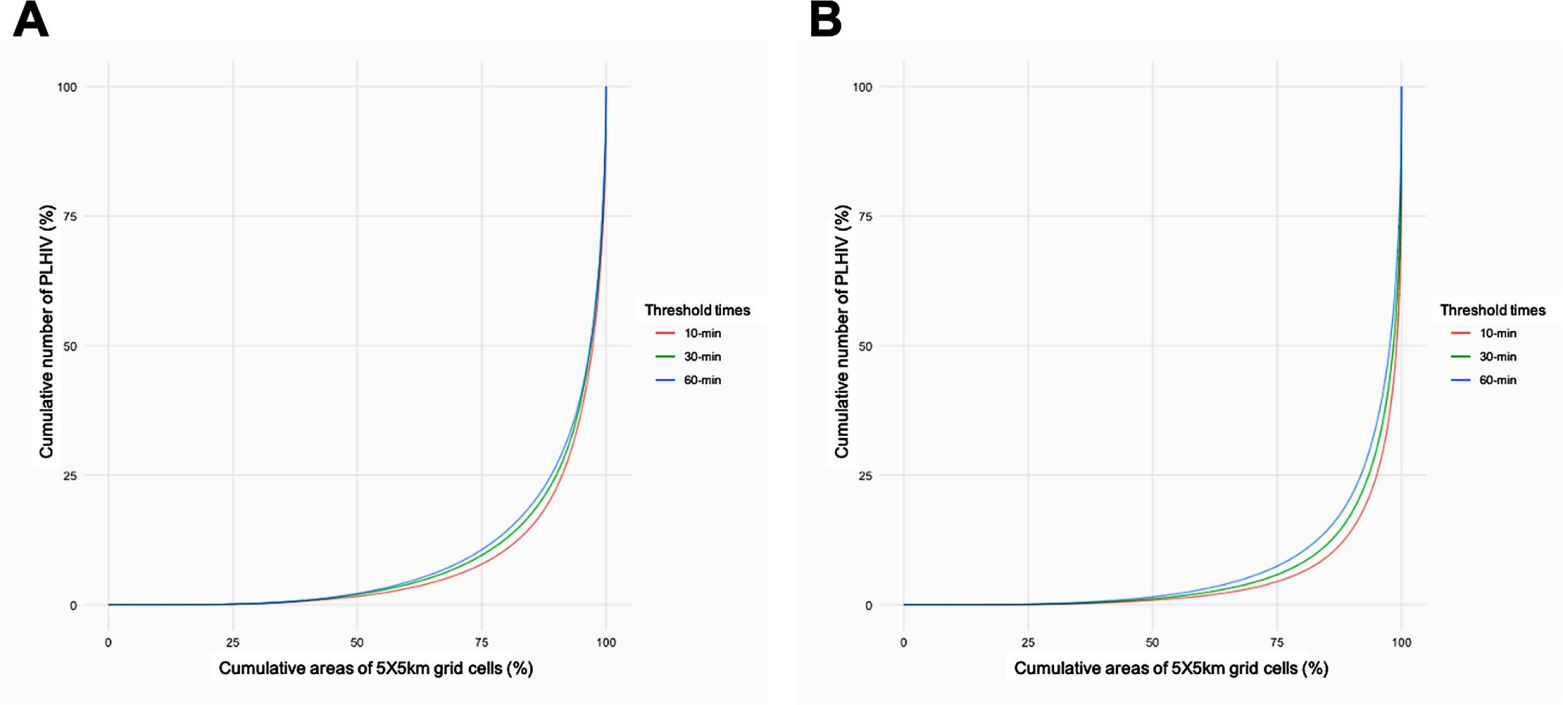
Lorenz curve showing the cumulative percentages of PLHIV compared to the cumulative percentages of 5X5km pixel. Curves showing the cumulative share of PLHIV using motorized (A) and non-motorized (B) transportation in underserved areas by each travel time threshold.

Fig 2 illustrates the Lorenz curves showing the cumulative percentages of PLHIV compared to the cumulative percentages of 5km x 5km pixel. A large proportion of PLHIV were concentrated in a small number of pixels with high densities of PLHIV (Fig 2A). Zero people lived with HIV in 55% of pixels in underserved areas at the 10-minute threshold (62.5% and 69.4% of pixels in underserved areas at the 30- and 60-minute thresholds, respectively). Less than 10 people lived with HIV in 87.9% of pixels in underserved areas at the 10-minute threshold (92.5% and 95.4% of pixels in underserved areas at the 30- and 60-minute thresholds, respectively). Lastly, less than 100 people lived with HIV in 99% of pixels in underserved areas at the 10-minute threshold (99.7% and 99.9% of pixels in underserved areas at the 30- and 60-minute thresholds, respectively).

Zimbabwe had the highest density of PLHIV in underserved areas at a single 5km x 5km pixel at the 10-minute threshold (more than 10,000 people), whereas Tanzania had the highest density of PLHIV at the 30-minute threshold (more than 4,000 people). Lastly, Nigeria had the highest density of PLHIV at a single 5km x 5km pixel at the 60-minute threshold (around 1,000 people).

**Fig 3:**
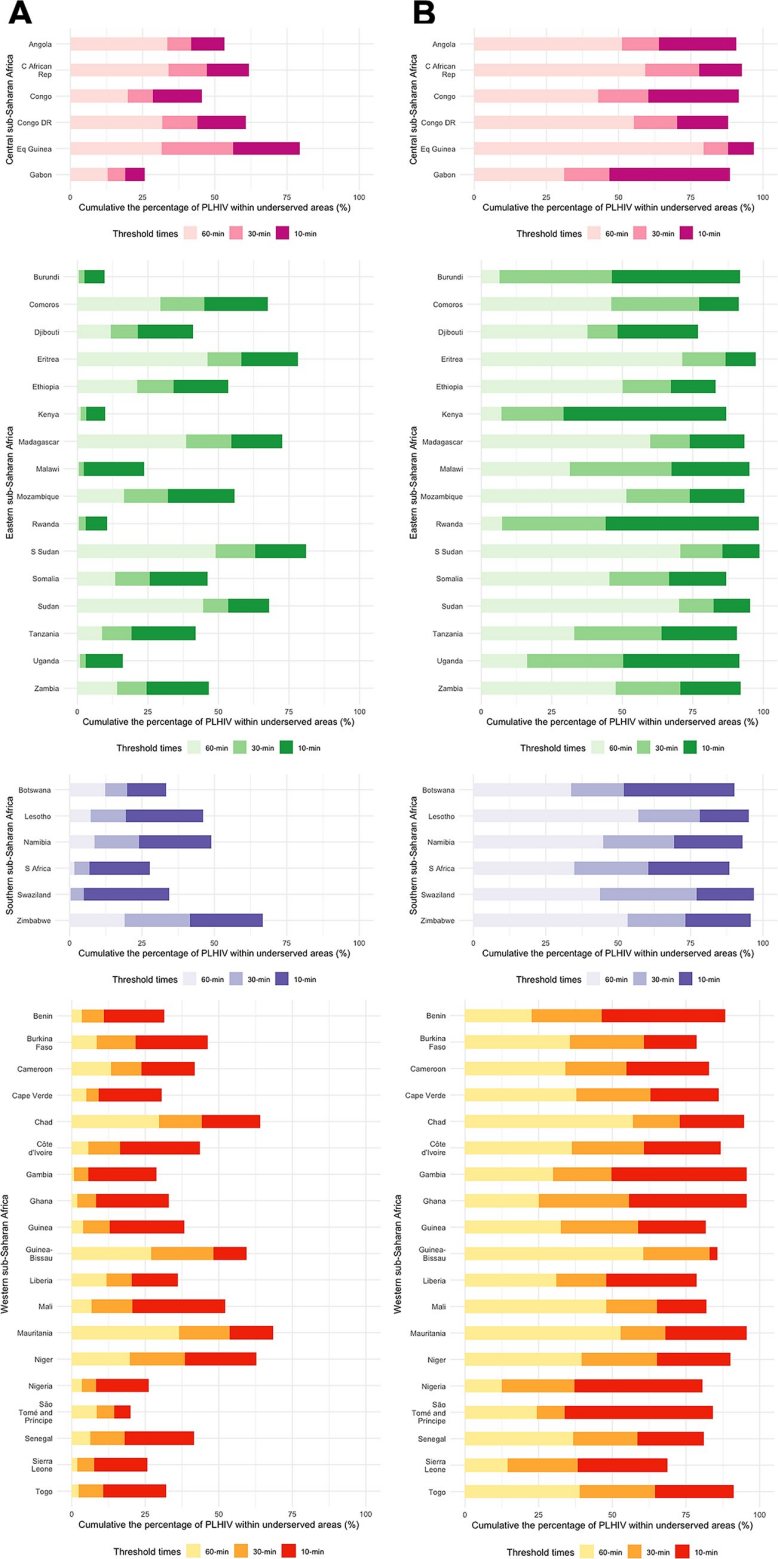
Cumulative bar graph of the percentages of People Living with HIV (PLHIV) in underserved areas with 10-, 30-, and 60-minute threshold times for each country by regions. The classification of regions refers to the study of the mapping HIV prevalence in sub-Saharan Africa [16]. (A. motorized; B. non-motorized transportation).

Fig 3A depicts the cumulative percentages of PLHIV in underserved areas in each country by region. An estimated 35% (≈ 7M) of the total PLHIV aged 15–49 years resided within underserved areas where access to a health care facility can be reached within 10 minutes by motorized transportation. South Sudan had the highest percent of PLHIV in underserved areas at all three thresholds (81%, 63%, and 48.9%, respectively) (Table 1). At the 10-minute threshold, 17 countries had more than 50% of the total PLHIV in underserved areas, ranging from 52% in Mali to 81% in South Sudan. Only three countries, Rwanda, Kenya and Burundi, had less than 15% of the total PLHIV in the country residing in underserved areas at the 10-minute threshold (10.4%, 9.8, and 9.6%, respectively), and had the lowest percentages of PLHIV at the 60-minute threshold (0.6%; 1.2%, and 0.4%, respectively). At the 60-minute threshold, only South Sudan and Sudan had more than 40% of PLHIV residing in underserved areas (48.9% and 44.5%, respectively). As the threshold time increased from 10 to 60 minutes, the percentages of PLHIV decreased in some countries such as Mali (ranging from 52.1% to 6.8%), Senegal (41.6% to 6.2%), Swaziland (34.5% to 0.3%), Gambia (28.8% to 0.8%), and Malawi (23.6% to 0.4%).

**Table 1:** The top five countries in sub-Saharan Africa with the highest number (%) of People Living with HIV (PLHIV) who cannot access health care facilities within thresholds by motorized transportation in 2017.

	COUNTRY	Estimated PLHIV	% of PLHIV
10-minute threshold			
	Total	7,374,229.40	100.00%
1	South Sudan	46,495.44	81.0%
2	Equatorial Guinea	28,644.77	79.4%
3	Eritrea	15,801.60	78.2%
4	Madagascar	21,698.55	72.6%
5	Mauritania	551.93	68.4%
30-minute threshold			
	Total	3,223,388.43	100.00%
1	South Sudan	36,128.90	63.0%
2	Eritrea	11,744.56	58.1%
3	Equatorial Guinea	20,316.65	56.3%
4	Madagascar	16,303.65	54.5%
5	Mauritania	432.99	53.7%
60-minute threshold			
	Total	1,543,848.53	100.00%
1	South Sudan	28,078.44	48.9%
2	Eritrea	9,301.03	46.0%
3	Sudan	31,682.98	44.5%
4	Madagascar	11,521.72	38.5%
5	Mauritania	293.92	36.4%

### PLHIV within underserved areas with non-motorized transportation (walking-only)

The walking-only results are more critical to people who have no access to or do not use motorized transportation. Fig 1D to 1F present the estimated number of PLHIV who use non-motorized transportation (walking-only) in underserved areas at the 10-, 30-, and 60-minute travel time thresholds by 5km x 5km pixel. Similar spatial patterns of PLHIV in underserved areas were observed for all thresholds, which showed a high density of PLHIV in south-eastern SSA. Specifically, the southern region had similar spatial patterns as that for PLHIV who use motorized transportation (Fig 1A–1C). The underserved areas based on a 10-minute walking time to the nearest health care facility across the region covered 99.5% of SSA (≈ 24M km^2^; Fig 1D), while 95.3% (Fig 1E) and 87.3% (Fig 1F) of underserved areas in SSA had 30 minute and 60 minute walking times, respectively. These areas corresponded to 88.7% (≈ 17.6M), 57.8% (≈ 11.5M), and 33.0% (≈ 6.6M) of PLHIV of the total population living with HIV aged 15–49 years, respectively. Descriptions of underserved areas at the country level can be found in S1 Text.

As the walking time threshold increased from 10 to 60 minutes, the average proportion of underserved areas with non-motorized transportation decreased from 99.6% to 88.2%. Specifically, underserved areas in Burundi, Rwanda, Sierra Leone, and Nigeria were significantly decreased from more than 90% to less than 50% (from 97.8% to 19.2%, from 98.5% to 25.9%, from 97.2% to 36.5%, and from 95.9% to 40.4%).

Lorenz curves indicated that at the 5km x 5km pixel level, PLHIV were concentrated in a small number of pixels with high density of PLHIV (Fig 2B), and the concave up curves increased at a lower rate of change than those for PLHIV using motorized transportation within underserved areas. Zero people lived with HIV in 50.7% of pixels in underserved areas at the 10-minute threshold (52.6% and 56.4% of pixels in underserved areas at the 30- and 60-minute thresholds, respectively). Less than 10 people lived with HIV in 83.7% of pixels in underserved areas at the 10-minute threshold (85.6% and 88.8% of pixels in underserved areas at the 30- and 60-minute thresholds, respectively). Lastly, less than 100 people lived with HIV in 97.6% of pixels in underserved areas at the 10-minute threshold (98.3% and 99.2% of pixels in underserved areas at the 30- and 60-minute thresholds, respectively). Mozambique had the highest density of PLHIV in underserved areas at a single 5km x 5km pixel at the 10- and 30-minute thresholds (more than 30,000 people) and Zambia had the highest density of PLHIV at a single 5km x 5km pixel at the 60-minute threshold (more than 8,000 people).

Fig 3B represents the cumulative percentages of PLHIV in underserved areas in each country by region. An estimated 88.7% (≈ 17.6M) of the total PLHIV aged 15–49 years resided within underserved areas where the closest health care facility can be reached within 10 minutes using non-motorized transportation. At the 10-minute threshold, more than 65% of PLHIV resided in underserved areas, and more than 90% of PLHIV lived in underserved areas in 27 countries. As the threshold time was increased from 10 to 60 minutes, the percentage of PLHIV decreased to less than 50% in two thirds of the countries in SSA (32 countries). However, some countries, such as Equatorial Guinea, Eritrea, South Sudan, and Sudan, contained more than 70% of PLHIV within underserved areas (Table 2).

**Table 2:** The top five countries in sub-Saharan Africa with the highest number (%) of People Living with HIV (PLHIV) who cannot access health care facilities within thresholds by non-motorized transportation in 2017.

	COUNTRY	Estimated PLHIV	% of PLHIV
10-minute threshold			
	Total	17,629,856.50	100.00%
1	South Sudan	56,589.56	98.6%
2	Rwanda	156,505.16	98.3%
3	Eritrea	19,663.33	97.2%
4	Swaziland	156,206.06	96.9%
5	Equatorial Guinea	34,905.07	96.8%
30-minute threshold			
	Total	11,477,993.72	100.00%
1	Equatorial Guinea	31,698.63	87.9%
2	Eritrea	17,521.20	86.7%
3	South Sudan	48,970.32	85.4%
4	Guinea-Bissau	24,413.26	83.1%
5	Sudan	58,645.11	82.3%
60-minute threshold			
	Total	6,562,127.10	100.00%
1	Equatorial Guinea	28,675.15	79.5%
2	Eritrea	14,407.65	71.3%
3	South Sudan	40,427.22	70.5%
4	Sudan	50,002.52	70.2%
5	Guinea-Bissau	17,822.63	60.6%

At the country level, Nigeria and Zimbabwe had significant spatiotemporal patterns as the threshold time increased. Nigeria, with the largest number of public hospitals in SSA [10], had the largest decrease in the size of underserved areas and percentages of the PLHIV as the threshold travel time increased from 10 to 60 minutes for both transportation modes (Fig 1A and 1C, and 1D and 1F). Within Nigeria, a high density of PLHIV was observed in the suburban area near Abuja and across Imo, Rivers, and Abia states within 10-minute travel time for both transportation modes (Fig 1A and 1D), and the density of PLHIV decreased as the travel time increased to 60-minute. With motorized transportation, less than 10% of PLHIV lived in the country near the border with Cameroon. Compared to previous estimates of HIV prevalence (2.8%), the prevalence of HIV in Nigeria decreased to 1.4% among adults aged 15–49 years even though the number of PLHIV was still high at 1.9 million people in 2018 [22].

In contrast, Zimbabwe had a moderate density of PLHIV within underserved areas in a 10-minute travel time (Fig 1A and 1D); however, the densities become noticeably higher as the travel time increased to 60 minutes for both transportation modes (Fig 1C and 1F). In the underserved areas with 10-minute threshold time, a high density of PLHIV was observed in Harare and near the city areas in Mashonaland Central, West, and East Provinces. However, the high-density areas expanded to almost all country territory as the threshold time increased to 60-minute, despite the served areas were excluded (Fig 1C and 1F). With motorized transportation, high-density areas were concentrated in Zimbabwe and some areas in Mozambique among the countries in SSA (Fig 1C). With non-motorized transportation, Zimbabwe is one of the high-density countries along with South Africa and Mozambique among SSA countries (Fig 1F). The density of PLHIV in a 10-minute threshold time with non-motorized transportation shows similar patterns to a previous study, with areas near Harare having most PLHIV [23].

## Discussion

The identified locations of underserved areas are an indicator of the challenge faced by PLHIV in accessing health services in SSA, a situation that is likely worsened by the COVID-19 pandemic. Our analyses provide geospatial insights towards the global target for 2025 by examining whether the current allocation of health facilities is sufficiently accessible to PLHIV in SSA, and how many PLHIV are underserved due to spatial heterogeneities of health care services.

Based on both motorized and non-motorized (walk only) transportation modes, we assessed the underserved areas in which the closest health care service is unreachable within desirable travel times (i.e., 10, 30, and 60 minutes) in SSA. A previous study suggested that the accessibility of health care services is sufficient in most of SSA, especially with motorized transportation [9]. However, our findings show that health care services with motorized transportation covers less than 10% of the total territory of SSA with 10-minute travel time (9.4%; ≈ 2.2M km^2^) and less than half of the total territory of SSA with 60-minute travel time (41.1%; ≈ 9.9M km^2^) in SSA. With non-motorized transportation, health care services cover 0.5% of the total territory of SSA with 10-minute travel time (≈ 0.1M km^2^) and only 12.7% of the total territory of SSA with a 60-minute travel time (≈ 3M km^2^). Specifically, we found substantial differences in the location and size of underserved areas between motorized and non-motorized transportation, and these differences increase with longer travel time window.

At the regional level, Western SSA countries show lower (less than 30%) percentage of PLHIV in underserved areas with 60-minute travel time to the closest health care facility, whereas the Central SSA countries show relatively high (more than 45%, except for Gabon) percentages. Among the Eastern SSA countries, while Burundi, Kenya, Rwanda, and Uganda show less than 20% of PLHIV in underserved areas with 60-minute travel time, Eritrea and South Sudan show more than 70% of PLHIV in underserved areas with 60-minute travel time. In general, some large countries, such as South Sudan, Mauritania, and Democratic Republic of the Congo, had larger underserved areas from health care facilities compared with smaller countries, such as Cape Verde, Rwanda, and Burundi. However, some large countries, such as Nigeria, South Africa, and Kenya, were notable exceptions with smaller underserved areas than those in smaller countries. These findings could offer insights into continental or regional levels of strategies to the world’s major health policymakers, such as the World Health Organization and World Bank, towards achieving goals such as the UNAIDS global target 2025 for the entire region.

One important function to routine treatment and management for PLHIV is an improvement of access to health care facilities with proper travel time. To cover geographically marginalized populations with clinical care, innovative targeting of health care services is required, including decentralization of HIV care. As the World Health Organization and UNAIDS emphasize the importance of antiretroviral therapy for all PLHIV [24, 25], high-resolution estimates of the location of underserved areas and PLHIV provide important information about the number of people who are potentially not receiving antiretroviral therapy. The presence of large underserved areas is a potential indicator of heterogeneously distributed hospitals. Furthermore, large underserved areas with high densities of PLHIV indicate not only longer travel times but also an insufficient number of hospitals and health care facilities.

SSA countries have faced a considerable shortage of hospitals and health workers, with more than 60% of countries having extreme shortages [26–28]. To overcome this challenge, although most studies for HIV intervention have focused on hospitals, decentralization and task shifting have been promoted as essential components of antiretroviral therapy scale-up [29, 30]. Many countries in SSA have decentralized HIV care from hospitals to health care facilities, including primary health care closer to communities [31]. The decentralization strategy seeks to reduce the travel distance of patients for improving access to care and retention in care, by expanding patients’ choice from physicians in hospitals to lower-level professionals in primary health care facilities for their antiretroviral therapy initiation and HIV management and treatment [32, 33]. Thus, decentralization promotes health care accessibility of patients living in regions with a limited number of hospitals, which is beneficial to people in low socioeconomic strata. In some SSA countries, including Malawi and South Africa, an antiretroviral therapy decentralization strategy has produced improved outcomes regarding retention and enrollment of patients and decrease lost to follow up in HIV care [34, 35]. There also has been a positive impact of antiretroviral therapy decentralization in rural areas where access to health care is more limited [32]. In Malawi, antiretroviral therapy decentralization resulted in decreased travel distance for treatment and increased retention in HIV care [32, 34]. The mean travel distance to hospitals decreased from 7.3 km to 4.7 km since antiretroviral therapy decentralization launched [32]. Moreover, the proportion of patients to attend hospital for HIV care increased from 89% to 99% since the program launched [32]. This study did not include primary health care to measure service areas from HIV care since the decentralization strategy has not covered all countries in Africa. However, the underserved areas detected in the study are likely to be in rural areas where there are low numbers of hospitals and doctors. Therefore, further research with regards to antiretroviral therapy retention from primary health care based on the decentralization strategy is needed.

Additionally, there are factors other than spatial accessibility, such as missed appointments, negative experiences at hospitals with staff, anticipated HIV stigma, discrimination, fear of disclosure and drug side effects, transportation costs, and need to obtain permission to travel (female only) are negatively associated with retention to HIV treatment [36, 37]. To overcome those constraints, besides the decentralization strategy, other solutions such as, launching mobile clinics or drones for delivering medication, or a representative of the community visits the hospital to get medicines for all PLHIV in the community can be considered [38–40].

Our PLHIV health care accessibility analysis and data are subject to several limitations. The completeness and accuracy of the health care facilities data are unknown [9]. Despite the data being the best available, it is possible that the datasets might contain incorrect facility locations, could be outdated as a consequence of new facilities opening and existing facilities closing, and there could be omissions such as when facility datasets are combined [9] since the health facility lists in Africa are from different sources. However, as it is the most complete record of public hospitals in SSA, and travel time from the friction surface is generally accurate [8, 9, 41], the dataset provides a considerable opportunity to estimate the underserved areas and travel times from health care facilities in SSA. In addition, we assumed that PLHIV travel to the nearest health care facility to measure underserved areas, which is not always the case [38]. If people do not visit their closest health care facility, the travel time will be longer and thus the size of underserved areas will be larger than our results. However, previous studies on measuring the relationship between travel distance and treatment initiation and retention showed that there is an opposite relationship between the two variables [32, 42]. The datasets that we used were generated in 2017, and therefore might not reflect current infrastructures, such as road systems or the built environment. The changes might affect accessibility and travel time within each pixel; thus, underserved areas based on the conditions of the built environment and road systems might be affected.

## Conclusion

Using geospatial approaches to measure underserved areas for PLHIV, we identified more than 7 million PLHIV with access to health care with a 10-minute travel time, and 1.5 million PLHIV in areas with a 60-minute travel time. Our study has noted the role of underserved areas as a potential source of HIV transmission, which suggests that targeted strategies to underserved areas could reduce not only the incidence of HIV but also other infectious disease, such as COVID-19. These findings can contribute to developing cost-effective policies for interventions aimed at underserved areas. Moreover, for areas where people are rarely likely to access HIV services, some alternatives could be suggested to improve accessibility, such as differentiated service delivery for HIV treatment or mobile outreach for HIV services. Further attention should be given to the development and implementation of tailored intervention programs in areas identified as underserved for PLHIV. The geospatial analysis we conducted in this study could be a complementary component of the decision-making process with stakeholders and the community.

## Supporting information

S1 Text Supporting methods and results. (DOCX)
