# Supplementary material for: When distance matters: Mapping HIV health care underserved communities in sub-Saharan Africa
Source: PLOS Glob Public Health. 2021 Nov 24;1(11):e0000013. doi: 10.1371/journal.pgph.0000013 (PMC10021734; doi:10.1371/journal.pgph.0000013)
Supplement: S1 Text — (DOCX) [file pgph.0000013.s001.docx]

**S1 Text for**

**When distance matters: Mapping HIV health care underserved communities in sub-Saharan Africa**

**Supplementary methods**

**Methods**

***Study area and data sources***

The estimated number of PLHIV for adults aged 15-49 years was generated following procedures, code, and data described in [1]. The estimated HIV prevalence was multiplied in each 5 km x 5km pixel across 47 countries in sub-Saharan Africa (SSA) by the corresponding population estimate from WorldPop [1, 2]. The gridded estimates of HIV prevalence were created by using a Bayesian spatiotemporal generalized linear mixed effects model. In order to capture possible nonlinear effects and complex interactions, 13 covariates were used for implementing an ensemble covariate model. The covariates are malaria incidence, night-time lights, prevalence of male circumcision, self-reported STI symptoms, marriage or living with a partner as married, one’s current partner living elsewhere, condom use at last sexual encounter, and multiple partners in the past year for men and for women, total population, travel time to the nearest settlement of more than 50,000 inhabitants, and urbanicity [1].

The locations of health care facilities were collected from Google Maps, Open Street Map, and recently published research about public health facilities in SSA [3]. The location of the public health facilities contains all public health care facilities and private-not-for-profit sectors managed by the government (e.g., the Ministry of Health, MoH), local authorities, and faith-based (FBO) and non-governmental organization (NGO) to represent public health services [3]. Friction surfaces represent optimal speed of travel computed based on the types of travel mode occurring within the pixel. The optimal speed is estimated by combining geographic characterized datasets, such as roads, railways, rivers, lakes, oceans, topographic conditions (e.g., slope and elevation), land cover types, and national borders except for air travel [4, 5].

***Mapping PLHIV in underserved areas***

Various travel times have been suggested as a threshold to access health care in countries in SSA and global estimates [4-12]. In this study, we used 10, 30, and 60 minutes thresholds that widely used in the country-level studies [5, 11, 12]. For example, a study of PLHIV in Uganda reports a median travel time of 30 minutes for PLHIV to access health care facility [12]. Another study for travel time to access primary health care in South Africa reported a median travel time of more than 80 minutes, and 65% of households travel more than 60 minutes for access to primary health care [11]. A recent study of the travel time to the nearest geolocated hospital or clinic across the world classified travel times as 10, 30, and 60 minutes to describe the number of populations within the travel times to health care facilities [4, 5].

**Supplementary Results**

***PLHIV within underserved areas with motorized transportation***

Table A presents the proportion of estimated underserved areas at the 10, 30, and 60 minute travel thresholds using motorized transportation in each country. Mauritania (99.4%) and Sudan (99.4%) had the highest proportion of underserved areas where health care services cannot be reached within 10 minutes, and 22 countries had more than 90% of their total territory as underserved areas. Burundi (21.5%) had the lowest proportion of underserved areas at the 10-minute threshold. Moreover, more than half of the countries in SSA (33 countries) had more than a 50% of the country territory designated as underserved areas at the 10-minute threshold. Burundi (9.2%) had the lowest proportion of underserved areas of the country territory at the 60-minute threshold. For this threshold, Sudan (93.3%) and Mauritania (91.2%) were the only countries with underserved areas covering more than 90% of the total country territory. Finally, underserved areas in Cape Verde (1.7%) and Swaziland (1.4%) covered less than 2% of the total country territory at the 60-minute threshold.

***PLHIV within underserved areas with non-motorized transportation (walking-only)***

Table B presents the proportion of estimated underserved areas at the 10, 30, and 60 minute travel thresholds for non-motorized transportation in each country. More than 99% of all study territories were identified as underserved areas with non-motorized transportation. At the 10-minute threshold, only two countries (Sao Tome and Principe and Comoros) had less than 95% of underserved areas within the country (94.4% and 93.8%, respectively (Fig 1E). At the 30-minute threshold, more than 90% of areas were identified as underserved areas in 36 countries. Burundi had the smallest size of underserved areas (63.2%). At the 60-minute threshold, eight countries had more than 95% of territories identified as underserved areas without access to motorized transportation (Sudan, Guinea-Bissau, Namibia, Mauritania, Republic of Congo, Chad, Equatorial Guinea, Angola, and Botswana). The proportion of underserved areas in four countries, namely Nigeria, Sierra Leone, Rwanda, and Burundi, had less than 50% of the total country territory identified as underserved to access to health care facilities. Notably, the underserved areas in Burundi covered less than 20% of the total country territory at the 60-minute threshold (19.2%).

**Supplementary tables**

**Table A. The top five country for the proportion of the estimated underserved areas within 10-, 30- and 60-minute thresholds by motorized transportation in each country in 2017.**

|  | **COUNTRY** | **Total estimated**  **underserved areas (km^2^)** | **% of the country territory** |
| --- | --- | --- | --- |
| 10-minute threshold | | | |
|  | Total | 21,853,402.2 | 100.00% |
| 1 | Mauritania | 1,038,861.9 | 99.4% |
| 2 | Sudan | 1,870,430.2 | 99.4% |
| 3 | Chad | 1,269,344.8 | 98.2% |
| 4 | Guinea-Bissau | 32,206.3 | 98% |
| 5 | Angola | 1,244,570.5 | 97.5% |
| 30-minute threshold | | | |
|  | Total | 17985897.2 | 100.00% |
| 1 | Sudan | 1819232.2 | 97.3% |
| 2 | Mauritania | 1001933.9 | 96.4% |
| 3 | Chad | 1159925.5 | 91.4% |
| 4 | Angola | 1118744.6 | 89.9% |
| 5 | Niger | 1055138.3 | 89.2% |
| 60-minute threshold | | | |
|  | Total | 14,208,223.5 | 100.00% |
| 1 | Sudan | 1,745,457.8 | 93.3% |
| 2 | Mauritania | 947,036.8 | 91.2% |
| 3 | Chad | 1,048,634.5 | 82.6% |
| 4 | Niger | 944,167.7 | 79.8% |
| 5 | Angola | 990,798.8 | 79.6% |

**Table B. The top five country for the proportion of the estimated underserved areas within thresholds by nonmotorized transportation in each country in 2017.**

|  | **COUNTRY** | **Total estimated**  **underserved areas (km^2^)** | **% of the country territory** |
| --- | --- | --- | --- |
| 10-minute threshold | | | |
|  | Total | 35,244,471.4 | 100.00% |
| 1 | Sudan | 1,869,737.2 | 100% |
| 2 | Chad | 1,267,814.0 | 99.9% |
| 3 | Mauritania | 1,038,015.0 | 99.9% |
| 4 | Namibia | 821,539.3 | 99.9% |
| 5 | Somalia | 629,563.4 | 99.9% |
| 30-minute threshold | | | |
|  | Total | 33,687,255.7 | 100.00% |
| 1 | Sudan | 1,865,493.8 | 99.7% |
| 2 | Guinea-Bissau | 32,049.0 | 99.5% |
| 3 | Namibia | 816,949.9 | 99.3% |
| 4 | Mauritania | 1,030,641.8 | 99.2% |
| 5 | Republic of Congo | 338,281.6 | 99.0% |
| 60-minute threshold | | | |
|  | Total | 30,788,850.4 | 100.00% |
| 1 | Sudan | 1,854,992.0 | 99.2% |
| 2 | Guinea-Bissau | 31,584.4 | 98.1% |
| 3 | Namibia | 804,401.6 | 97.8% |
| 4 | Mauritania | 1,010,494.0 | 97.3% |
| 5 | Republic of Congo | 331,271.5 | 97.0% |

**References**

1. Dwyer-Lindgren L, Cork MA, Sligar A, Steuben KM, Wilson KF, Provost NR, et al. Mapping HIV prevalence in sub-Saharan Africa between 2000 and 2017. Nature. 2019;570(7760):189-93. doi: 10.1038/s41586-019-1200-9.

2. Project W. WorldPop Project 2014. Available from: <www.worldpop.org>.

3. Maina J, Ouma PO, Macharia PM, Alegana VA, Mitto B, Fall IS, et al. A spatial database of health facilities managed by the public health sector in sub Saharan Africa. Scientific Data. 2019;6(1):134. doi: 10.1038/s41597-019-0142-2.

4. Weiss DJ, Nelson A, Gibson HS, Temperley W, Peedell S, Lieber A, et al. A global map of travel time to cities to assess inequalities in accessibility in 2015. Nature. 2018;553(7688):333-6. doi: 10.1038/nature25181.

5. Weiss D, Nelson A, Vargas-Ruiz C, Gligorić K, Bavadekar S, Gabrilovich E, et al. Global maps of travel time to healthcare facilities. Nature Medicine. 2020:1-4.

6. Alegana VA, Maina J, Ouma PO, Macharia PM, Wright J, Atkinson PM, et al. National and sub-national variation in patterns of febrile case management in sub-Saharan Africa. Nature communications. 2018;9(1):1-7.

7. Falchetta G, Hammad AT, Shayegh S. Planning universal accessibility to public health care in sub-Saharan Africa. Proceedings of the National Academy of Sciences. 2020;117(50):31760-9.

8. Wigley A, Tejedor-Garavito N, Alegana V, Carioli A, Ruktanonchai CW, Pezzulo C, et al. Measuring the availability and geographical accessibility of maternal health services across sub-Saharan Africa. BMC medicine. 2020;18(1):1-10.

9. Ouma PO, Maina J, Thuranira PN, Macharia PM, Alegana VA, English M, et al. Access to emergency hospital care provided by the public sector in sub-Saharan Africa in 2015: a geocoded inventory and spatial analysis. The Lancet Global Health. 2018;6(3):e342-e50. doi: 10.1016/S2214-109X(17)30488-6.

10. Hulland E, Wiens K, Shirude S, Morgan J, Bertozzi-Villa A, Farag T, et al. Travel time to health facilities in areas of outbreak potential: maps for guiding local preparedness and response. BMC medicine. 2019;17(1):1-16.

11. Tanser F, Gijsbertsen B, Herbst K. Modelling and understanding primary health care accessibility and utilization in rural South Africa: An exploration using a geographical information system. Social Science & Medicine. 2006;63(3):691-705. doi: <https://doi.org/10.1016/j.socscimed.2006.01.015>.

12. Akullian AN, Mukose A, Levine GA, Babigumira JB. People living with HIV travel farther to access healthcare: a population-based geographic analysis from rural Uganda. J Int AIDS Soc. 2016;19(1):20171. Epub 2016/02/13. doi: 10.7448/ias.19.1.20171. PubMed PMID: 26869359; PubMed Central PMCID: PMCPMC4751409.
